# Treatment failure and drug resistance in HIV-positive patients on tenofovir-based first-line antiretroviral therapy in western Kenya

**DOI:** 10.7448/IAS.19.1.20798

**Published:** 2016-05-25

**Authors:** Katherine Brooks, Lameck Diero, Allison DeLong, Maya Balamane, Marissa Reitsma, Emmanuel Kemboi, Millicent Orido, Wilfred Emonyi, Mia Coetzer, Joseph Hogan, Rami Kantor

**Affiliations:** 1 Division of Infectious Diseases, Alpert Medical School, Brown University, Providence, RI, USA; 2 Academic Model Providing Access to Healthcare (AMPATH), Eldoret, Kenya; 3 Department of Medicine, School of Medicine, College of Health Sciences, Moi University, Eldoret, Kenya; 4 Center for Statistical Sciences, Brown University, Providence, RI, USA; 5 Department of Biostatistics, School of Public Health, Brown University, Providence, RI, USA

**Keywords:** HIV, drug resistance, tenofovir, Kenya, subtype

## Abstract

**Introduction:**

Tenofovir-based first-line antiretroviral therapy (ART) is recommended globally. To evaluate the impact of its incorporation into the World Health Organization (WHO) guidelines, we examined treatment failure and drug resistance among a cohort of patients on tenofovir-based first-line ART at the Academic Model Providing Access to Healthcare, a large HIV treatment programme in western Kenya.

**Methods:**

We determined viral load (VL), drug resistance and their correlates in patients on ≥six months of tenofovir-based first-line ART. Based on enrolled patients’ characteristics, we described these measures in those with (prior ART group) and without (tenofovir-only group) prior non-tenofovir-based first-line ART using Wilcoxon rank sum and Fisher's exact tests.

**Results:**

Among 333 participants (55% female; median age 41 years; median CD4 336 cells/µL), detectable (>40 copies/mL) VL was found in 18%, and VL>1000 copies/mL (WHO threshold) in 10%. Virologic failure at both thresholds was significantly higher in 217 participants in the tenofovir-only group compared with 116 in the prior ART group using both cut-offs (24% vs. 7% with VL>40 copies/mL; 15% vs. 1% with VL>1000 copies/mL). Failure in the tenofovir-only group was associated with lower CD4 values and advanced WHO stage. In 35 available genotypes from 51 participants in the tenofovir-only group with VL>40 copies/mL (69% subtype A), any resistance was found in 89% and dual-class resistance in 83%. Tenofovir signature mutation K65R occurred in 71% (17/24) of the patients infected with subtype A. Patients with K65R had significantly lower CD4 values, higher WHO stage and more resistance mutations.

**Conclusions:**

In this Kenyan cohort, tenofovir-based first-line ART resulted in good (90%) virologic suppression including high suppression (99%) after switch from non-tenofovir-based ART. Lower virologic suppression (85%) and high observed resistance levels (89%) in the tenofovir-only group impact future treatment options, support recommendations for widespread VL monitoring in such resource limited settings to identify early treatment failure and suggest consideration of individualized resistance testing to design effective subsequent regimens.

## Introduction

In 2014, 10.7 million people in sub-Saharan Africa received antiretroviral therapy (ART), an effort that reduced the number of AIDS-related deaths in the region by 48% [1]. As new evidence accumulates on early ART benefits, the number of treated patients will surely increase [2,3].

Current World Health Organization (WHO)-recommended first-line ART includes tenofovir (TDF), efavirenz (EFV) and XTC (lamivudine=3TC or emtricitabine=FTC), with zidovudine (AZT) and nevirapine (NVP) as alternatives for TDF and EFV, respectively [4]. TDF has replaced both stavudine (d4T) and AZT given its favourable toxicity profile, improved dosing and cost-effectiveness [4,5]. However, consequences of TDF use are still to be determined in resource-limited settings (RLS), in which viral load (VL) monitoring is limited, immunological monitoring has high misclassification rates of treatment failure and non-B HIV-1 subtypes predominate [6,7]. The TDF signature reverse transcriptase (RT) resistance mutation K65R, which decreases susceptibility to all nucleoside RT inhibitors (NRTIs) except AZT, has been found to vary by subtype and geographical region upon failure of a TDF-containing first-line regimen: 0 to 6% in subtype A (Europe), 0 to 17% in subtype B (USA and Europe), 12 to 70% in subtype C (South Africa), and 57 to 59% in subtype G and circulating recombinant form (CRF) 02_AG (Nigeria) [8–16]. Potential mechanisms for this variability, in addition to limited VL monitoring in RLS, include the homopolymeric stretch of adenosines immediately preceding K65 in subtype C and subtype-specific regimen efficacy [17–20].

In Kenya, where adult HIV prevalence is 6.1%, 63% of medically-eligible patients receive ART and infection is diverse with predominant subtypes A (50–80%), D (10–20%) and C (5–15%) [21,22]. Few studies on acquired resistance, prior to TDF incorporation in first-line regimens, report first-line resistance in 13 to 94% [23–26], with low (13%) K65R levels [25]. Transmission of this mutation in Kenya is rare [27].

The Academic Model Providing Access to Healthcare (AMPATH) is a major HIV programme in western Kenya, treating >140,000 patients [28]. As recommended by WHO, AMPATH guidelines now include TDF in first-line regimens upon treatment initiation and as a less-toxic option for patients on formerly recommended first-line regimens. While patients and their physicians may choose to continue their current regimen, TDF use is expected to increase in both treatment-naïve and -experienced patients on first-line therapy – a trend recently reported from other regions in Kenya [29]. To evaluate the impact and consequences of treatment guideline changes and to examine the prevalence of treatment failure and resistance at enrolment, we performed a cross-sectional study of prospectively recruited AMPATH clinic patients on TDF-based first-line ART. This study design permits inclusion of patients with and without prior exposure to non-TDF-based first-line regimens.

## Methods

### Study setting and design

This cross-sectional study was conducted at Moi Teaching and Referral Hospital (MTRH) clinic, AMPATH's largest, managing 26,791 adults [28]. HIV-positive patients were enrolled between December 2012 and November 2013 as they came to their routine clinic visit. Inclusion criteria included: (1) ≥18 years, (2) WHO-recommended first-line ART ≥6 months (TDF+3TC/FTC+EFV/NVP) and (3) self-reported adherence >50% (past month and past seven days). After providing informed consent, participants were interviewed and underwent phlebotomy for CD4, VL and drug resistance testing. Interviews and chart reviews provided demographic information and HIV-related measures, including gender, age, self-reported adherence, WHO stage, co-morbidities, ART history, medication change indications, prior pregnancy and previous CD4 counts. At the time of the study, monitoring of patients on ART included six monthly CD4 count and targeted VL testing upon suspected treatment failure based on immunological/clinical WHO guidelines. Lifespan and Moi University ethics committees approved the study.

### Laboratory methods

CD4 (FACSCaliber system; Becton Dickenson, San Jose, CA) and VL (Amplicor; Roche Molecular, Pleasanton, CA) testing were done at the Good Clinical Laboratory Practice (GCLP) compliant AMPATH reference laboratory, which participates in the United Kingdom National External Quality Assessment Service and Rush University Viral Quality Assurance programmes and is accredited for ISO 15189 by Kenya Accreditation Services. Confirmatory VL results after adherence counselling, as recommended by WHO guidelines, were not available.

Genotyping was performed on all patients with detectable (>40 copies/mL) VL. Frozen plasma was shipped to the United States for genotyping. RNA was extracted from 200 µL of plasma via Biomerieux's MiniMAG (Biomerieux, Durham, NC). To increase assay sensitivity of samples with VL <1000 copies/mL, RNA was extracted from 400 uL plasma. An in-house genotyping assay was used to generate a 1300-bp fragment including the RT gene. Briefly, a nested RT-PCR was performed using SuperScript III One-Step RT-PCR (Life Technologies, Carlsbad, CA, USA), followed by second-round PCR using Platinum Taq DNA Polymerase High Fidelity (Life Technologies) per manufacturer's instructions. Cycling parameters for RT-PCR were 45°C for 45 minutes, 95°C for 2 minutes, followed by 40 cycles of 94°C for 15 seconds, 55°C for 20 seconds and 72°C for 2 minutes, followed by a 10-minute hold at 72°C. Cycling parameters for PCR were 35 cycles of 94°C for 15 seconds, 55°C for 20 seconds and 72°C for 2 minutes, followed by a 10-minute hold at 72°C. PCR products were Sanger sequenced at the Rhode Island Genomics and Sequencing Center and assembled with Sequencher v4.10.1 [30]. PCR and sequencing primers have been previously described [31]. Sequences were submitted to Genbank (accession numbers KU900868 – KU900902).

### Data analysis

**Figure 1:**
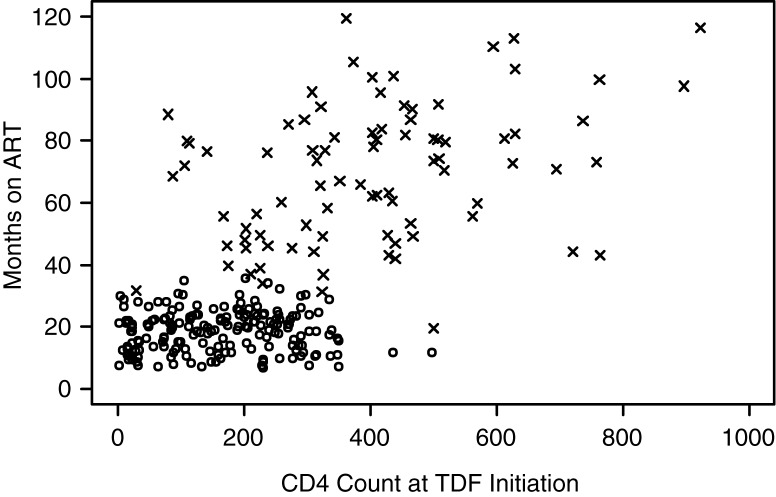
**Distributions of time on treatment versus CD4 count at TDF initiation in the two patient groups.** Distributions of months on ART (Y axis) versus CD4 count at TDF initiation (X axis) in the TDF-Only (circles) and prior ART (X's) patient groups are shown. CD4 data are available for 266/333 participants.

The primary objective of the study was to evaluate virologic failure and drug resistance rates among patients on TDF-containing first-line ART. As described below, we observed during recruitment that participants meeting enrolment criteria fit into one of two distinct groups: those who began ART on a TDF-containing regimen (tenofovir-only group) and those who switched to TDF from a prior non-TDF first-line regimen (i.e. AZT/d4T+3TC/FTC+EFV/NVP; prior ART group). Analyses were therefore designed to (1) describe virologic failure and resistance in each group, (2) explore predictors of failure and resistance in the two groups as the data and sample sizes allow, and (3) examine HIV-1 subtype and associated potential genotypic pathways and mutation correlation for the development of resistance (including specifically K65R). We do not attempt a thorough comparison of the two groups because they differ substantially by key variables such as time on first-line ART and CD4 count at TDF initiation, and consequently standard adjustments for confounding are not feasible (see Figure 1).

Two definitions for treatment failure were considered: VL>40 copies/mL (assay detection limit) and VL>1000 copies/mL (WHO threshold). Genotyping quality control and distance calculations were performed with SQUAT [32]. Resistance interpretation and predicted susceptibilities were derived with Stanford database tools [33]. Intermediate-high predicted resistance was considered clinically significant. Subtyping was done with REGA v3 [34]. Fisher's exact and Wilcoxon rank sum tests were used to compare demographic and clinical covariates of several patient subgroups: those with and without VL>40 copies/mL stratified by group; and those with and without K65R, stratified by group, among participants with genotypes.

To evaluate potential impacts of subtype-specific codon usage on K65R selection, we compared nucleic acids at codons 64 to 66 in subtypes A, B, C and D sequences from ART-naïve or TDF-treated patients, derived from: (1) Stanford database (33) and (2) AMPATH (prior and current studies) [22].

## Results

### Patient enrolment

Inclusion criteria were met by 352 patients over the recruitment period who were approached for possible enrolment. Eighteen patients were excluded because of ineligible medication history (*n=*10), missing clinical data (*n=*6), poor adherence (*n*=2); and one declined participation.

Table 1 shows demographic, clinical and laboratory data of the 333 enrolled participants. Of this cohort, 55% were female, median age was 41 years, and median CD4 336 cells/µL. Through self-report, 94 and 98% were fully adherent in the past month and week, respectively. Over half (60%) had WHO stage 3 or 4, 5% had prior non-tuberculosis (TB) opportunistic infections (OIs), and 21% previously had TB. Five women had prior pregnancies, two received non-TDF-based ART and three were on ART before pregnancy. At enrolment, 60% of the patients were on 3TC/TDF/NVP and 40% on 3TC/TDF/EFV. Median time on treatment was 25 months, and median time on TDF 21 months. Twelve patients had treatment interruptions, nine during TDF therapy (median 33 days, range 13–123), and three between non-TDF- and TDF-based regimens (durations of 8, 1723 and 1878 days).

**Table 1:** Demographic clinical and laboratory data of enrolled patients according to study group^a^

	Total			TDF-only group			Prior ART group		
			
	Total (*n*=333)	VL≤40 (*n*=274)	VL>40 (*n*=59)	Total (*n*=217)	VL≤40 (*n*=166)	VL>40 (*n*=51)	Total (*n*=116)	VL≤40 (*n*=108)	VL>40 (*n*=8)
Female	184 (55%)	149 (54%)	35 (59%)	117 (54%)	87 (52%)	30 (59%)	67 (58%)	62 (57)	5 (62%)
Age (years)^c^	41 (23, 82)	42 (27, 72)	38 (23, 82)	40 (23, 82)	41 (27, 72)	38 (23, 82)	44 (27, 66)	44 (27, 64)	39 (33, 66)
1-month non-adh									
None	314 (94%)	258 (94%)	56 (95%)	206 (95%)	157 (95%)	49 (96%)	108 (93%)	101 (94%)	7 (88%)
Some (<50%)	19 (6%)	16 (6%)	3 (5%)	11 (5%)	9 (5%)	2 (4%)	8 (7%)	7 (6%)	1 (12%)
1-week non-adh									
None	326 (98%)	268 (98%)	58 (98%)	213 (98%)	162 (98%)	51 (100%)	113 (97%)	106 (98%)	7 (88%)
Some (<50%)	6 (2%)	6 (2%)	0 (0%)	4 (2%)	4 (2%)	0 (0%)	2 (2%)	2 (2%)	0 (0%)
Most (>50%)	1 (0%)	0 (0%)	1 (2%)	0 (0%)	0 (0%)	0 (0%)	1 (1%)	0 (0%)	1 (12%)
TDF Rx^c^									
3TC, TDF, NVP	201 (60%)	163 (59%)	38 (64%)	120 (55%)	87 (52%)	33 (65%)	81 (70%)	76 (70%)	5 (62%)
3TC, TDF, EFV	132 (40%)	111 (41%)	21 (36%)	97 (45%)	79 (48%)	18 (35%)	35 (30%)	32 (30%)	3 (38%)
CD4 count (cells/mL)^b^,^c^,^d^	336 (5, 1043)	349 (44, 1043)	211 (5, 869)	298 (5, 1035)	326 (64, 1035)	191.5 (5, 869)	426 (44, 1043)	426 (44, 1043)	486.5 (250, 773)
CD4%^b^,^c^,^d^	21 (1, 48)	23 (6, 48)	14 (1, 48)	19 (1, 48)	21 (6, 48)	12 (1, 48)	24 (7, 47)	24 (7, 47)	25.5 (11, 37)
TB Ever	69 (21%)	58 (21%)	11 (19%)	48 (22%)	38 (23%)	10 (20%)	21 (18%)	20 (19%)	1 (12%)
WHO stage^d^									
1	81 (24%)	72 (26%)	9 (15%)	59 (27%)	53 (32%)	6 (12%)	22 (19%)	19 (18%)	3 (38%)
2	53 (16%)	41 (15%)	12 (20%)	38 (18%)	27 (16%)	11 (22%)	15 (13%)	14 (13%)	1 (12%)
3	143 (43%)	116 (42%)	27 (46%)	86 (40%)	61 (37%)	25 (49%)	57 (49%)	55 (51%)	2 (25%)
4	56 (17%)	45 (16%)	11 (19%)	34 (16%)	25 (15%)	9 (18%)	22 (19%)	20 (19%)	2 (25%)
Months on ART^c^	25.2 (6.9, 119.3)	26.6 (7, 119.3)	20.6 (6.9, 95.5)	20.1 (6.9, 50.7)	20.4 (7, 50.7)	18.4 (6.9, 35)	72.3 (19.5, 119.3)	72.3 (19.5, 119.3)	67 (43.1, 95.5)
Months on TDF^c^	20.8 (6.5, 68.4)	21.1 (6.5, 68.4)	19.1 (6.9, 35)	20.1 (6.9, 50.7)	20.4 (7, 50.7)	18.4 (6.9, 35)	23.8 (6.5, 68.4)	23.8 (6.5, 68.4)	25.5 (11.1, 29.6)
Months prior ART^c^				-	-	-	46.6 (0.9, 98.2)	45.5 (0.9, 98.2)	49.1 (15.1, 71.2)
Any OI^e^	17 (5%)	13 (5%)	4 (7%)	11 (5%)	7 (4%)	4 (8%)	6 (5%)	6 (6%)	0 (0%)
TI									
During TDF^c^	9 (3%)	5 (2%)	4 (7%)	9 (4%)	5 (3%)	4 (8%)	0 (0%)	0 (0%)	0 (0%)
Prior to TDF	3 (1%)	2 (1%)	1 (2%)	-	-	-	3 (3%)	2 (2%)	1 (12%)
SE at switch^f^									
Lipodystrophy							81 (70%)	76 (70%)	5 (62%)
Anaemia							11 (9%)	11 (10%)	0 (0%)
Neuropathy							7 (6%)	7 (6%)	0 (0%)
Combined^g^							2 (2%)	1 (1%)	1 (12%)
Not indicated							15 (13%)	13 (12%)	2 (25%)
VL>1000 copies/mL^c^	34 (10%)	0 (0%)	34 (58%)	33 (15%)	0 (0%)	33 (65%)	1 (1%)	0 (0%)	1 (12%)

a Values are presented as median (range) for continuous variables and n (%) for categorical variables

b CD4 counts and % were missing for three patients

c Indicates variables that were significantly different (*p<*0.05) between TDF-only group and prior ART group at baseline

d Indicates variables that were significantly associated (*p<*0.05) with failure in the TDF-only group

e While on TDF-based therapy

f Refers to the side effects documented at the time the prior ART group patients were switched from prior first-line ART to TDF-containing ART

g Combined category refers to patients who were switched from prior first-line ART to TDF-containing ART for both anaemia and neuropathy.

TDF, Tenofovir; EFV, Efavirenz; NVP, Nevirapine; ART, antiretroviral treatment; TB, tuberculosis; adh, adherence (specific questions included: “All patients miss HIV medications sometimes. How many times did you miss your medications in the past seven days/month?”); OIs, opportunistic infections; Rx, Regimen; SE, side effects; TI, treatment interruption.

Of 333 participants, 217 had no ART exposure before TDF-based regimens (TDF-only group), and 116 had prior non-TDF-based first-line ART exposure (prior ART group; 96% exposed to d4T, 23% to AZT, 35% to EFV and 79% to NVP). Gender, adherence, WHO staging and TB history were similar between groups. Compared to the prior ART group, TDF-only group patients were younger (median 40 years vs. 44, *p=*0.014), had lower median CD4 values (298 vs. 426 cells/µL, *p<*0.001; 19% vs. 24%, *p*≤0.001), were on overall ART and TDF-based ART for substantially less time (*p*≤0.0001) and were less likely to be on NVP-based regimens (55% vs. 70%, *p=*0.01). Most patients in the prior ART group had documented side effects upon switch to TDF-containing regimens, including lipodystrophy (70%), anaemia (9%), neuropathy (6%) and both anaemia and neuropathy (2%); (reason unavailable for 13%).

### Treatment failure

Of 333 patients, 59 (18%) had detectable VLs and 34 (10%) had VL>1000 copies/mL (Table 1). The median age and the median CD4 count of patients with detectable VL (59% female) were 38 years and 211 cells/µL (14%), respectively, at enrolment; 64% were in WHO stage 3 or 4, 7% had a history of non-TB OIs and 19% had a history of TB.

Patients in the TDF-only group failed TDF-based therapy at higher rates than the prior ART group by both VL thresholds (24% vs. 7% had detectable VL; 15% vs. 1% had VL>1000 copies/mL). Treatment failure in either group was not significantly associated with age, gender, adherence, OIs, specific non-NRTI (NNRTI) exposure or time on ART, which could be due to small sample sizes.

Among the TDF-only group, participants failing treatment were more likely to have advanced WHO stage (67% Stage 3 or 4 vs. 52% in the suppressed group, *p=*0.029) and lower CD4 values (192 vs. 326 cells/µL; 12% vs. 21%, *p<*0.0001 for both). With only eight failures in the prior ART group, ascertaining correlates of failure was difficult; however, failure was marginally associated with seven-day ART adherence (98% perfect adherence in the suppressed group vs. 88% in failures, *p=*0.07).

It is not feasible to compare rates of viral failure between the groups because the distributions of two important confounders, duration of ART treatment and CD4 count at TDF initiation (available for 266/333 participants), showed little to no overlap (Figure 1).

### HIV diversity and drug resistance

Sequences were available from 35 of 51 patients with detectable VL from the TDF-only group and two of eight patients from the prior ART group. The 22 patients (16 from the TDF-only group and six from the prior ART group) whose genotypes were not available were treated for a median of 21 months (IQR 17–41) and had a median VL of 111 copies/mL (IQR 70–344, with two samples above 35,000 copies/mL). In univariate analysis, successful genotyping was significantly associated with higher VL. Due to the few sequences from the prior ART group, genotypic comparisons with the TDF-only group were not performed. Detailed resistance results for the TDF-only group are provided in Table 2. The median age of the 35 patients was 37 years; 60% were female and median CD4 count was 128 cells/µL. Eighty-six percent (30/35) had VL>1000 copies/mL after a median of 15 months on TDF; 83% were on 3TC/TDF/NVP and 17% on 3TC/TDF/EFV; subtype diversity included 69% subtype A, 11% C, 11% D and 9% AD recombinants.

**Table 2:** Mutations found in TDF-only patients with plasma sequences

Study ID^a^	Subtype	Viral load (copies/mL)	Last NNRTI backbone^c^	NRTI mutations^d^	NNRTI mutations	Months on treatment
1	A	1,351,982	EFV	D67G, K70Q, M184V	K101P, K103S, E138Q	8
2	A	92,081	EFV	**K65R**, M184V	A98G, V179F, Y181C, G190A	14
3	A	337,468	EFV	**K65R**, K70T, M184I	V90I, L100I, K103N	22
4	A	31,683	EFV	**K65R**, M184I	V90I, K103N, M230L	27
5	A	287,657	EFV	**K65R**, V75M, Y115F, M184V	K103N, V108I, Y181C, G190A	13
6	A	297,459	NVP	A62AV, **K65R**, M184V	K101E, Y181C, G190A	14
7	A	3825	NVP	M184V	G190A	24
8	A	87,827	NVP	K70E, Y115F, M184V	Y181C, H221Y	23
9	A	100,342	NVP	**K65R**, M184I	Y181C, M230L	9
10	A	3516	NVP	A62AV, **K65R**, Y115FY, M184V	A98AG, K103KN, Y181C	23
11	A	8247	NVP	**K65R**, M184MV	V90I, Y181G, G190A	15
12	A	89	NVP	None	K103N	13
13	A	41	NVP	None	None	21
14^b^	A	15,888	NVP	A62V, **K65R**, D67N, M184V, K219E	K101EK, E138AT, Y181C, G190A	15
15	A	4983	NVP	M184I	K103N	25
16	A	47,280	NVP	None	None	12
17	A	150,649	NVP	**K65KR**, D67DG, M184MV	K101E, Y181CY, G190A	33
18	A	65,519	NVP	**K65R**, M184V	Y181C	10
19	A	1541	NVP	**K65R**, M184I	V90I, V108I, Y181C	24
20	A	7001	NVP	**K65R**	K103N, Y181C	18
21	A	9476	NVP	**K65R**, Y115F, M184V	K103N, Y181C	15
22^b^	A	58,286	NVP	M41L, **K65KR**, L74V, M184V	K101E, Y181C, G190A	12
23	A	199,751	NVP	**K65R**, M184V	V108I, Y181C, H221Y	11
24	A	127,716	NVP	**K65R**, Y115FY	Y181C, H221Y	11
25	C	38,437	EFV	A62V, **K65R**, M184V	V106M, Y181C, G190S	29
26	C	60,147	NVP	**K65R**, Y115F, M184V	K101E, Y181C, G190A	24
27	C	5585	NVP	A62AV, **K65KR**, K70EK, M184V	A98G, Y181C, G190A	14
28^b^	C	972	NVP	**K65KR**, D67DN, K70R, M184V, K219E	A98G, G190A	28
29	D	1,175,462	EFV	K70E, M184V	Y181C	10
30	D	7968	NVP	**K65R**, M184V	K101P, K103N	31
31	D	939,458	NVP	**K65R**, M184V	K101E, Y181CFGV, G190AG	13
32	D	153	NVP	None	V106A	30
33^b^	DA	7592	NVP	**K65R**, M184V, K219EK	K103N, Y181C	28
34	AD	45	NVP	None	None	12
35	AD	12,652	NVP	None	None	21

a Underlined study ID indicates mutation patterns not found in the Stanford Database

b Patients with K65R in whom TAMs (underlined mutations) were also found

c In addition to 3TC +TDF for all patients

d K65R mutations in bold.

RT resistance was detected in 89% (31/35) of patients, 89% (31/35) to NNRTIs, 83% (29/35) to NRTIs and 83% (29/35) to both. Of the 31 patients with any resistance mutation, 1/31 (3%) had one-class and 34/35 (97%) had dual-class resistance. Overall 155 mutations were detected (78 NRTI, 76 NNRTI and one major PI), with a median of five mutations per patient (range 0–9), two NRTI (range 0–5) and two NNRTI-associated (range 0–4). Of five patients with VL<1000, two had no resistance, two had one NNRTI-associated mutation and one had multiple NRTI-associated mutations.

**Figure 2:**
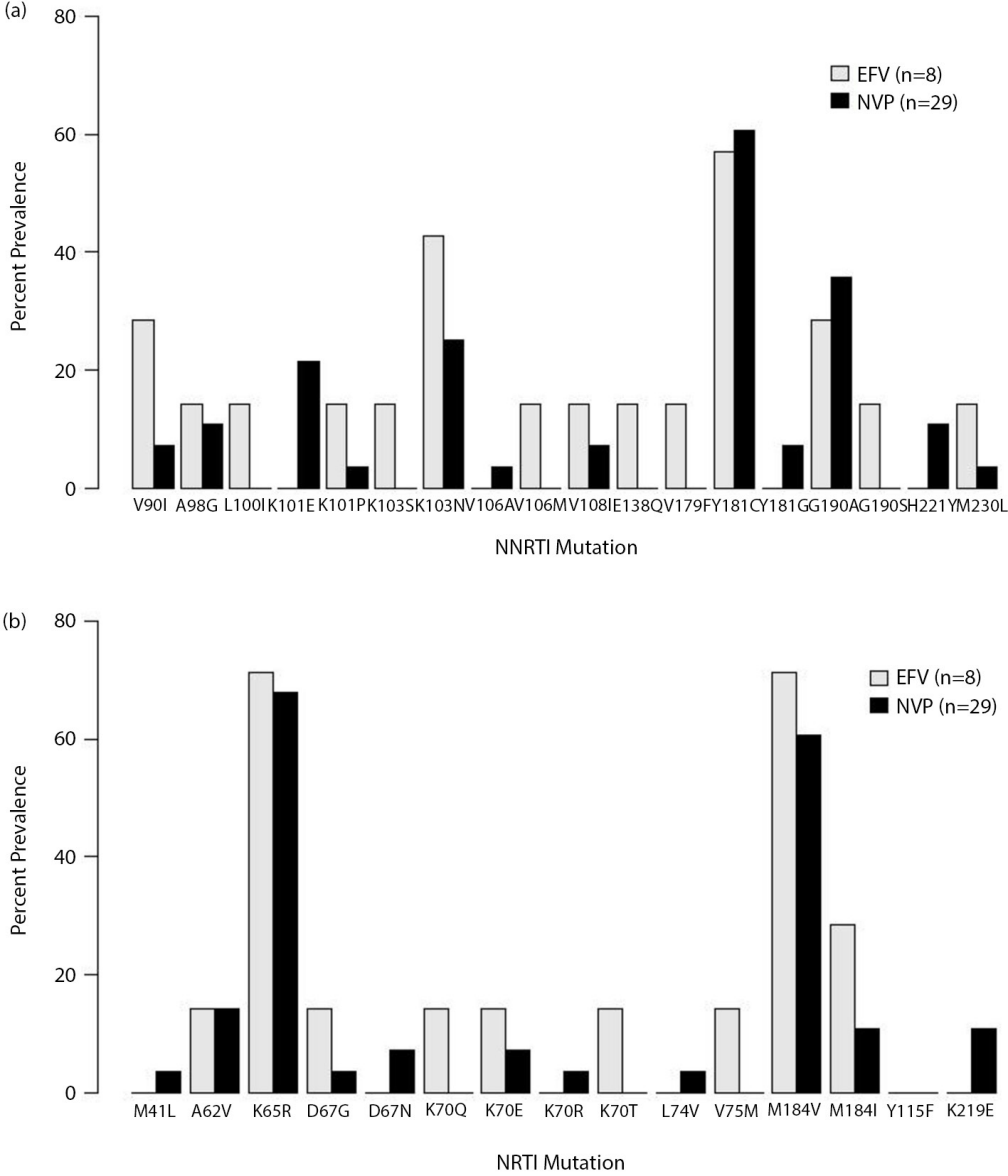
**Prevalence of NRTI and NNRTI resistance mutations by NNRTI backbone used in first-line regimens.** Figure depicts the prevalence of NNRTI (a) and NRTI (b) mutations by use of EFV as NNRTI backbone (light grey) or NVP as NNRTI background (black).

K65R developed in 69% (24/35) of patients (Figure 2): 17/24 (71%) patients with subtype A, 4/4 (100%) subtype C, 2/4 (50%) subtype D and 1/3 (33%) AD recombinants (*p=*0.223). Compared to those without K65R, those with this mutation had lower median CD4 values (74 versus 269, *p=*0.019; 7% vs. 18%, *p=*0.006), more advanced WHO stage (79% stage 3–4 vs. 18%, *p=*0.004) and more RT mutations (96% ≥2 vs. 73%, *p=*0.001). There was no significant association between K65R development and specific NNRTIs (5/7, 71% with K65R on EFV vs. 19/28, 68% with K65R on NVP), age, subtype, or time on treatment. Only 4/24 patients with K65R had any IAS-USA-defined thymidine analogue mutations (TAMs) [35]. Mutations associated with K65R included Y181C (19/24, 79%, with K65R vs. 2/11, 18%, without; *p=*0.002); G190A/S (12/24, 50%, with K65R vs. 1/11, 9%, without; *p=*0.027); and G190A/Y181C co-occurrence (9/24, 38% with K65R vs. 0/11 without; *p=*0.033).

Other common (>20%) RT mutations were NRTI-associated M184V/I (27/35, 77%) and NNRTI-associated Y181C/G/Y (22/35, 63%), G190A/S (13/35, 37%) and K103N/S (11/35, 31%). TDF-associated K70E was found in 3/35 (9%) patients, none with TAMs. Examination of specific mutations by NNRTI backbone revealed overall similarity (Figure 2). Compared to sequence data available from the Stanford Database, 18 of 31 (58%) study sequences with resistance mutations had mutation patterns that were unique and not seen among any subtype, and 26/31 (84%) were unique among the same subtype (Table 2; underlined study IDs).

**Figure 3:**
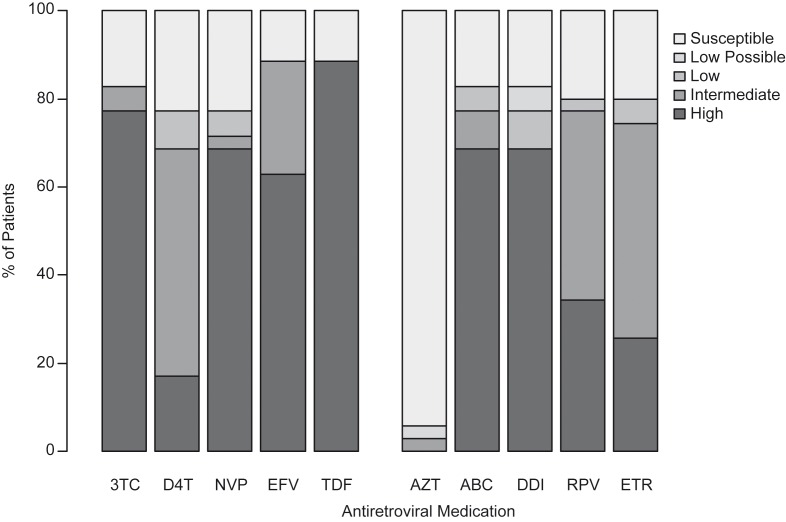
**Predicted drug resistance based on Stanford Database of all patients with sequences in the TDF-only group.** This graph demonstrates the percent of TDF-Only Patients with one of five resistance categories (as defined by the Stanford Database) to each antiretroviral medications. Medications are divided by currently used options (left) and future options. 3TC, lamivudine; ABC, abacavir; AZT, zidovudine; D4T, stavudine; DDI, didanosine; FTC, emtricitabine; TDF, tenofovir; EFV, efavirenz; ETR, etravirine; NVP, nevirapine; RPV, rilpivirine.

Intermediate-high resistance to first-line ART drugs was seen in 89% of the patients (31/35), including 83% (29/35) to 3TC and FTC, 71% (25/35) to TDF, 86% (31/35) to EFV and 89% (31/35) to NVP (Figure 3). Intermediate-high resistance to future ART options was seen in 77% of patients (27/35), including 77% (27/35) to etravirine (ETR) and rilpivirine (RPV) and 77% (27/35) to abacavir (ABC). Though the vast majority of patients (33/35, 94%) remained fully susceptible to AZT, one had potential low-level (ID 6, Table 2) and another had intermediate (ID28) resistance to it, neither with prior exposure.

### Codon analysis

**Figure 4:**
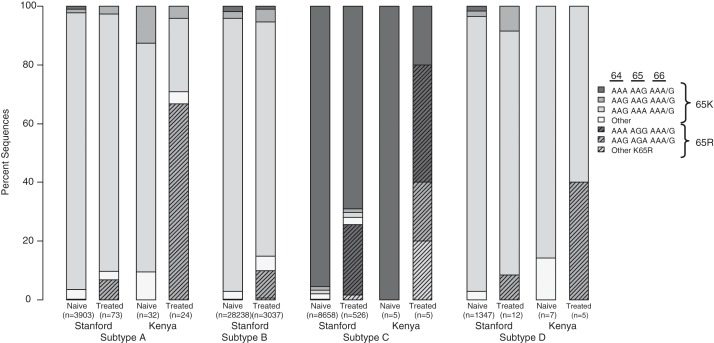
**Codon analysis of ART-naïve and TDF-treated patients with HIV-1 subtypes A, B, C and D.** This figure demonstrates sequence diversity at RT codons 64, 65 and 66 in ART naïve and TDF-treated patients with subtypes A, B, C and D. Bars are organized according to (a) subtype, (b) source of sequence (Stanford Database vs. sequences from our studies at AMPATH in Kenya), (c) ART naïve vs. TDF treated. Striped bars represent sequences with TDF-associated mutation K65R. Darkest gray bars (both solid and striped) represent sequences with poly-adenosine template. AMPATH sequences from both the TDF-only and prior ART group were included in this figure.

Available sequence data at RT positions 64–66 were compiled from 45,794 patients from the Stanford Database (42,146 naïve – 3,903 subtype A, 28,238-B, 8,658-C, 1,347-D; 3,648 TDF-treated – 73-A, 3,037-B, 526-C, 12-D); and 78 AMPATH patients (44 naïve: 32-A, 5-C, 7-D; 34 TDF-treated: 24-A, 5-C, 5-D) (Figure 4). The most common codons of the wild type amino acid lysine (K) at position 64 were similar among subtypes A (AAG 96%), B (AAG 95%) and D (AAG 96%), and differed from subtype C (AAA 96%) in treatment-naïve and -experienced patients from both Stanford and AMPATH. The poly-adenosine AAA codon at position 64, thought to promote K65R development in subtype C, was present in only 1.0% of subtype A naive (1% Stanford, 0% AMPATH) and 1.7% of subtype D (1.7% Stanford, 0% AMPATH) sequences. Equally, the most common codons of the mutated amino acid arginine (R) at RT position 65 were similar among subtypes A (AGA in 100%), B (AGA in 96%) and D (AGA in 100%), and differed from subtype C (AGG in 96%) in treatment-experienced patients from both Stanford and AMPATH. Codons at position 66 were highly concordant among all subtypes.

## Discussion

To our knowledge, this is the largest report of HIV-positive Kenyans failing first-line TDF-based ART, highlighting three main findings. First, defining virologic failure as VL>1000 copies/mL, 99% of those switching to TDF and 85% of those starting on TDF-based first-line ART had virologic suppression after a median of 25 months, demonstrating good virologic response among 333 AMPATH adults infected mostly with HIV-1 subtypes A, C and D. Second, switch from non-TDF- to TDF-based first-line therapy was successful and associated with low treatment failure. Third, among failing patients, high levels of resistance were observed across subtypes, with high K65R rates posing particular concern. Our results demonstrate public health consequences of the implementation of first-line TDF-containing regimens in RLS and support switching to TDF-based regimens from other first-line medications. These data highlight the importance of virologic monitoring to avoid resistance accumulation upon treatment failure and suggest consideration of resistance testing for individual care in RLS.

The proportions of study participants failing TDF-containing ART (18% detectable VL and 10% VL>1000) are within the range of published data, mostly from US and European clinical trials [19]. The lower treatment failure rate among the prior ART group (1%>1000 copies/mL) compared to the TDF-only group (15%) probably reflects the fact that they had been engaged in care for longer, likely associated with their improved adherence to treatment and success of prior first-line regimens. These likely explanations are strengthened by the higher CD4 values of this group at study enrolment and by the finding that most switches from first to second line were due to drug toxicity rather than treatment failure, further representing high treatment motivation. These results should reassure clinicians in RLS to switch to TDF-based first-line ART, despite prior concerns of K65R development in patients with prior d4T exposure [36]. Follow-up of those patients who were switched from non-TDF- to TDF-based first-line ART and subsequently to second-line ART was not part of this study and is needed to fully examine this possibility. Though failure and K65R development were not associated with NNRTI backbone as previously suggested [19], such associations should continue to be investigated given our small sample-size.

This study provides new data on TDF use in diverse subtypes. The high overall (89%) and dual-class (83%) resistance is consistent with other reports from AMPATH [23,26] and other RLS upon any (not solely TDF-based) first-line ART [37]. The few studies that evaluated resistance in TDF-based first-line regimens in RLS, mostly in subtypes C, G and CRF02_AG from Southern Africa and Nigeria, do not report overall resistance but estimate NRTI- (94 to 100%) and NNRTI-associated resistance (57 to 97%) to be high [9–11,13,16]. Though mutation frequencies in this study, other than K65R, were similar to prior reports [13], unique mutation patterns in 58% of the patients and specific mutation co-occurrence support the need for continued research on subtype-specific resistance, particularly with changing treatment guidelines [38].

K65R is a TDF signature mutation conferring resistance to all NRTIs but AZT, is rarely transmitted [27], is less common in subtype B-infected patients failing therapy [15], and is variable in RLS [9–11,13,16]. The high barrier to K65R development as reported in resource-rich settings may reflect high potency of K65R-selecting drugs, significant viral fitness constraints and unique RNA structural considerations [20]. Our data offer new information on the interplay between subtype and K65R. We, for the first time, report high K65R occurrence (71%) upon TDF-based first-line failure in subtype A, a globally prevalent subtype and the most common in Kenya. These data augment prior contradicting reports from Europe, one reporting low K65R rates in subtype A compared to other subtypes [12] and another reporting no inter-subtype difference [8]. Although very small in numbers, these data also support high K65R occurrence in subtype C (100%, 4/4) [9,13] and introduce its high occurrence in subtype D (50%, 2/4). According to our analysis of subtypes A and D from Stanford and AMPATH, these high K65R rates are not explained by the adjacent poly-A template, as proposed for subtype C [20]. Although pre-treatment samples from study participants were not available for examination of codon usage, the availability of sequences from ART-naïve AMPATH patients with a similar subtype distribution strengthens this observation.

Several considerations are relevant to the demonstrated increased K65R levels. First, our data support the reported association between K65R, Y181C and G190A, suggesting synergistic fitness effects, and extend it to NVP (not only EFV) treatment [39]. Second, lack of TAMs and no prior TAM-associated medication exposure may facilitate K65R development [39]. Lastly and importantly, limited VL monitoring is likely contributory because delayed diagnosis of failure can lead to increased resistance [37]. In fact, reports in subtype C from settings in which virologic monitoring is routine, including South Africa, demonstrate lower K65R rates [11]. The lack of routine virologic monitoring at AMPATH at the time of this study likely played a role in K65R development, as evidenced by indicators of advanced disease like low CD4, high WHO stages, more RT mutations and potentially failing therapy for longer.

Our results therefore support implementation of routine VL monitoring for earlier identification of treatment failure and consideration of individual resistance testing to optimize patient care. The latter may be particularly true for K65R detection because its presence would indicate the need to switch to AZT and its absence would indicate the possibility to recycle TDF in a subsequent first- or second-line regimen. In fact, in this study, 31% of the patients had no K65R, some after as many as 33 months of TDF-based first-line ART. Switching such patients to AZT would be premature. The one patient with enough accumulated resistance to both TDF and AZT is concerning and further justifies the need for VL and resistance testing. Such consequences are important for public health considerations, which should include improvement of treatment monitoring by increased VL testing resulting in less drug-resistance accumulation.

The major study limitation was a small sample size, resulting in low power to examine some associations with failure and resistance, and inability to compare failure and resistance patterns between groups and subtypes. In addition, traditional Sanger sequencing may have underestimated K65R and other minority resistant variants; we were unable to follow the prior ART group from ART start or switch to TDF; VL failure was not confirmed; adherence was self-reported; time of failure was not quantified; and lastly, enrolled participants were on TDF for various time periods and those lost to follow-up were not included, potentially underestimating actual failure rates. Nevertheless, the study represents real-life clinical care circumstances and provides new needed data from RLS.

## Conclusions

In this study from a large HIV treatment programme in western Kenya, patients’ response to a TDF-based first-line ART was good and switch from non-TDF-based ART was successful. High prevalence of resistance, in particular K65R, was observed, impacting future treatment options. Results support recommendations for widespread VL monitoring in RLS to identify early treatment failure, and the need to consider individualized resistance testing to accurately design subsequent regimens [40]. Even in settings with limited treatment options as in RLS, the impact of available drug resistance testing on clinical decisions may differ, from adherence intensification and medication recycling to a change to conventional second-line or the need for third-line options. Moreover, though currently low, the impact of K65R transmission in such settings on first-line regimens, as well as on pre-exposure prophylaxis (PrEP) with TDF-containing regimens, is yet to be determined.
